# Nurses’ perspectives on user-friendly self-sampling interventions for diagnosis of sexually transmitted infections among young women in eThekwini district municipality: a nominal group technique

**DOI:** 10.1186/s12913-023-10353-6

**Published:** 2024-01-18

**Authors:** Ziningi N. Jaya, Witness Mapanga, Boitumelo Moetlhoa, Tivani P. Mashamba-Thompson

**Affiliations:** 1 School of Health Systems and Public Health, Faculty of Health Sciences, University of Pretoria, Pretoria, South Africa; 2 Department of Biomedical Science, Faculty of Natural Science, Mangosuthu University of Technology, KwaZulu-Natal, South Africa; 3 Faculty of Health Sciences, University of Pretoria, Pretoria, South Africa

**Keywords:** Barriers, Sexually transmitted infections, Self-sampling, Strategies, eHealth solutions

## Abstract

**Background:**

Syndromic management in the main non-laboratory-based management approach for sexually transmitted infections (STI) in most low- and middle-income countries (LMICs) but it has limitations. Self-sampling has been proven as a suitable alternative approach to help improve management STIs by improving access to diagnosis among vulnerable populations. We sought to determine health workers’ perspectives on user-friendly self-sampling interventions for STIs among young women in eThekwini District Municipality.

**Methods:**

Healthcare workers providing STI healthcare services in the study location participated in a nominal group technique (NGT) workshop. The NGT workshop was aimed enabling collaboration with key health providers in identifying user-friendly self-sampling interventions for diagnosis of STIs among young women. Data collection was conducted in two phases: phase 1 determined barrier that hinder young women from accessing current STI healthcare services and phase 2 focused on determining the key strategies for self-sampling interventions to diagnose STIs in young women. Thematic analysis and percentage form analysis were used to examine qualitative and quantitative data respectively.

**Results:**

The following barriers were identified: negligence; myths about STIs; fear of judgement; denial; operating hours; lack of knowledge of STI symptoms and safe sex practices; and stigma associated with STIs. The following strategies were suggested: hand out self-sampling kits at popular restaurants; collect self-sampling kits from security guard at primary healthcare clinics (PHCs); receive STI diagnostic results via SMS or email or the clinic for treatment; improve youth friendly services at PHCs; educate the public on proper use of the kits. Education about STIs and handing out self-sampling kits at clinics, universities, schools, pharmacies or via outreach teams were ranked high priority strategies.

**Conclusions:**

The findings highlight the need to address stigma and fear of judgment and provide comprehensive education to improve healthcare-seeking behaviour in young women. Additionally, the study also indicates that using eHealth solutions could significantly enhance the accessibility and efficiency of STI healthcare services in LMICs.

## Background

The burden of sexually transmitted infections (STIs) remains a major public health challenge throughout the globe [1, 2], and the associated mortality and morbidity remain high [3]. Effective management of STIs is essential to reduce the burden of infections and associated complications. However, in many parts of the globe, including low-and-middle-income-countries (LMICs), healthcare infrastructure is often limited in providing quality affordable STI healthcare services [4]. The current research explores and presents barriers faced by young women residing in underserved communities in accessing STI healthcare services and identifies strategies for self-sampling interventions to improve access.

Diagnosis and management of STIs often relies on syndromic management, particularly in resource-limited settings due to cost implications [5]. This approach involves treating patients based on their clinical presentation instead of specific laboratory diagnoses [6–8]. While it has proven valuable and effective in treating and managing STIs, it presents several limitations. The limitations include failure to identify asymptomatic infections and thus prevent timely diagnosis and treatment of infected individuals [6, 9]. Furthermore, since the issuing of treatment is not based on confirmed laboratory diagnoses, it often leads to over-diagnosis and over-treatment of patients which increases costs of healthcare and development of antibiotic resistance [5]. Several researchers have reported on the growing trend of drug resistant gonorrhoea and chlamydia [10, 11]. As such, there is a dire need for a paradigm shift from syndromic management to pathogen specific treatment of STIs to mitigate the limitation of syndromic management.

In light of these challenges, self-sampling for STI diagnosis has emerged as a promising alternative to syndromic management [12, 13]. Self-sampling allows people to collect their won biological specimens at a convenient location of their choice [13] and then the specimens are used for laboratory diagnosis of STIs. Self-sampling not only addresses the issues of stigma and lack of privacy [12, 14, 15] which are presented by syndromic management, but it also has the potential to improve access to STI healthcare even for individuals in resource-limited areas [16]. Furthermore, it is an effective alternative to screen for STIs, including asymptomatic infections where patients may not necessarily seek medical assistance [17, 18]. Despite its popular uptake in high-income countries (HICs), self-sampling remains an investigative intervention in LIMCs. The affordability of self-sampling kits and practicability of delivering results are some of the factors that require careful consideration to effectively adopt self-sampling interventions in LMICs. However, these factors we not explored in this study.

We utilised a nominal group technique (NGT) to engage healthcare workers from primary healthcare clinics (PHCs) in underserved communities in eThekwini District Municipality in KwaZulu-Natal. The high prevalence of STIs in this province [19] made it a suitable study area because study findings would benefit many people at risk of STIs in this province. Through the NGT approach healthcare workers from diverse PHCs and backgrounds came together to identify barriers to young women accessing STI healthcare services. Subsequently, the healthcare workers collaborated to develop strategies for self-sampling to address the identified barriers. Considering that syndromic management is the current approach to treating and managing STIs in South Africa, the barriers to STIs being referred to in this study were in relation to symptomatic infections. This paper presents the findings of this NGT which includes identified barriers and strategies developed. By understanding the barriers and identifying strategies to mitigate barriers, we aim to contribute towards the development of a user-friendly self-sampling intervention easily accessible to young women in underserved communities.

### The significance of involving healthcare workers

Healthcare professionals including nurses, medical doctors, and other staff involved in STI healthcare service provision are uniquely positioned to provide insight into the challenges of providing this service. They often serve as intermediaries between healthcare facilities and the communities which they serve promoting a patient-centred approach to healthcare service provision. As such, their involvement in this NGT is significant because they hold the necessary knowledge and expertise to identify barriers and develop strategies for self-sampling to improve access to STI healthcare. By engaging them we tapped into their collective knowledge and expertise to bridge the gap between real-life experiences at PHCs and research related to STI healthcare services. Contributions from our group of healthcare professionals were expected to yield strategies contextually relevant to STI healthcare nuances. Ultimately, their contribution led to the development of strategies grounded in the experiences of both the healthcare professionals and their patients.

## Methods

STI healthcare service providers from PHCs located in underserved urban communities in eThekwini District Municipality were invited to participate in a co-creation workshop. This co-creation workshop was aimed towards identifying barriers that prevent young women from accessing STI healthcare services. It also aimed to identify key strategies for a self-sampling intervention to improve access to STI healthcare services for young women in underserved communities.

### Sampling

Study participants were recruited directly from selected PHCs in eThekwini District Municipality. STI healthcare service providers that included nurses and medical doctors were requested to participate in the study. Despite rigorous recruitment strategies and issues of availability of medical staff, a total of eight nurses participated on the NGT. The following eligibility criteria were utilised to select eligible study participants:

- Inclusion criteria
  - Healthcare workers working in PHCs located in underserved communities in eThekwini district municipality.
  - Healthcare workers involved in STI healthcare service provision at PHCs in underserved communities in the selected district municipality.
- Exclusion criteria
  - All healthcare personnel who reported no direct involvement in STI healthcare service provision.
  - Healthcare workers from PHCs outside of those in underserved communities in the selected district municipality.

#### Challenges with participant recruitment and scheduling of NGT during sampling

The recruitment of study participants and scheduling of the NGT workshop presented challenges that were unexpected. As a result, data collection was delayed. The following is an account of the challenges experienced and the action taken to address them:

- Participant recruitment
  Clinic management personnel at the PHCs were welcoming and open to accommodate the research team for data collection. Communication about the study was shared with the relevant healthcare personnel through the clinic manager. However, regardless of the process followed, there were numerous instances when staff was unavailable to speak to the research team because the PHCs were busy with a large number of patients. In those instances, the research team would be told to wait until the relevant healthcare workers were available. Initially a total of 16 participants were recruited for the NGT and promised their participation. However, in the end only eight participants were available for the NGT for various reasons. The reasons included not being able to get time off from patient consultations due to staff shortages, and being on sick leave. Ultimately, only eight nurses involved in STI healthcare participated in the NGT.
- Scheduling of NGT
  The PHCs from which participants were recruited were far from each other and this presented a challenge with scheduling of the NGT at a time and day suitable for all. The situation was further exacerbated by protest action at one of the clinics on a day on which NGT was scheduled. Since the research team could not access PHCs nor research the staff, the NGT had to be rescheduled. Although the NGT date changed several times, the NGT was eventually conducted.

### Study design

The NGT is a highly regarded qualitative exploratory research method that merges idea generation and problem-solving within the context of group dynamics [20, 21]. This method operates through organized small group discussions, typically involving a cohort of 6 to 12 participants [22], with NGTs being optimal when consisting of five to nine participants [23, 24]. The application of this methodology is well-documented both within the healthcare and other research domains [25, 26]. It serves as an instrument in identifying pivotal attributes for effectively implementing Discrete Choice Experiments (DCEs) [27]. One unique attribute of the NGT approach is its capacity to foster the generation of diverse ideas while concurrently cultivating consensus among participants [25, 28]. This capability, which is transformative, is a direct result of its capacity to promote active engagement from all participants, even individuals who may be apprehensive and withhold their perspectives [25]. By being so inclusive, NGT mitigates the potential for dominant individual voices to overshadow the process of knowledge creation. This ensures that a wide range of multiple responses are received in response to the question posed.

The NGT method consists of three pivotal stages, namely silent generation, round-robin, and ranking of all contributions made. Silent generation is the initial stage where participants take time to think about their responses to a question that has been posed. During this stage participants contemplate and commit their insights to writing. Subsequently, the round-robin sharing stage follows where individual contributions are systematically revealed, documented, and discussed for clarification where necessary. This stage facilitates a robust exchange of ideas. Finally, all contributions are systematically ranked based on their perceived priority to each participant. This process renders the NGT method, rigorous and versatile to improve the quality of any research it informs.

### Procedure

We conducted an NGT workshop on the 6^th^ of February 2023 by collaborating with consenting healthcare workers involved in STI healthcare service provision at the selected PHCs [29]. The eight participants were split into two groups of four members. The NGT was conducted in two consecutive phases to address 2 questions. The participants were asked the following questions:

- Question 1 (for phase 1): What are the barriers that prevent or limit young women from accessing current STI healthcare management services?
- Question 2 (for phase 2): What would be the key strategies to deliver self-sampling for STI diagnosis among young women to mitigate the barriers to access?

In phase 1 the main focus was to determine barriers that hinder young women from accessing current STI healthcare services. Phase 2 mainly focused on determining the key strategies that can be employed to efficiently deliver self-sampling interventions for STI diagnosis in young women. Each phase was conducted in the following stages:

- Phase 1: the healthcare workers were requested to share their knowledge about barriers that prevent young women from utilising the existing STI healthcare services. The PI (ZNJ) and facilitator instructed the stakeholders to independently group their contributions according to themes and present them to the entire group of participants. Thereafter ZNJ, listed the themes in a voting form to enable the study participants to independently rank each theme according to a level of importance. The level of importance was determined using a ranking score between 1 to 7 where “1” represents a low priority barrier and “7” represents a high priority barrier.
- Phase 2: in this stage stakeholders were asked to identify key strategies for delivering STI self-sampling to mitigate the previously outlined barriers to access. Thereafter all the stakeholders independently grouped their contributions into themes and presented them to the entire group of participants. Once again, ZNJ listed the themes in a voting form to enable voting through ranking. The ranking score was between 1 to 7 where “1” represented a low priority strategy and “7” represents a high priority strategy.

Following the NGT workshop, ZNJ compiled a report of the NGT proceedings and shared it with the stakeholders for comments.

### Data management and analysis

Quantitative data collected during the ranking step was calculated in phase 1 as a total importance score for each barrier by adding individual participant scores. However, in phase 2 a total importance score of each strategy was calculated to signify the perceived efficacy of the suggested strategies for self-sampling to diagnose STIs. Thematic content analysis was utilised to analyse qualitative data to identify themes that emerged from data that was presented during the NGT discussions. To limit researcher bias through numerous theoretical perspectives and predetermined ideas, coding categories were directly extracted from data text.

## Results

A total of eight key stakeholders from two clinics namely, Cato Manor Clinic and Sydenham Clinic agreed to participate in our study. All the participants were female nurses and employed at PHCs. Three nurses were aged between 25 to 35. Four nurses were aged between 36-45, and only one was between the age of 46 – 55 years. The characteristics of all stakeholders are outlined in Table 1 below.

**Table 1:** Characteristics of stakeholders

Age group in years	Number of nurses	PHC of employment
25 – 35	1	Sydenham Heights Clinic
	2	Cato Manor Clinic
36—45	2	Sydenham Heights Clinic
	2	Cato Manor Clinic
46—55	1	Sydenham Heights Clinic

**Fig. 1:**
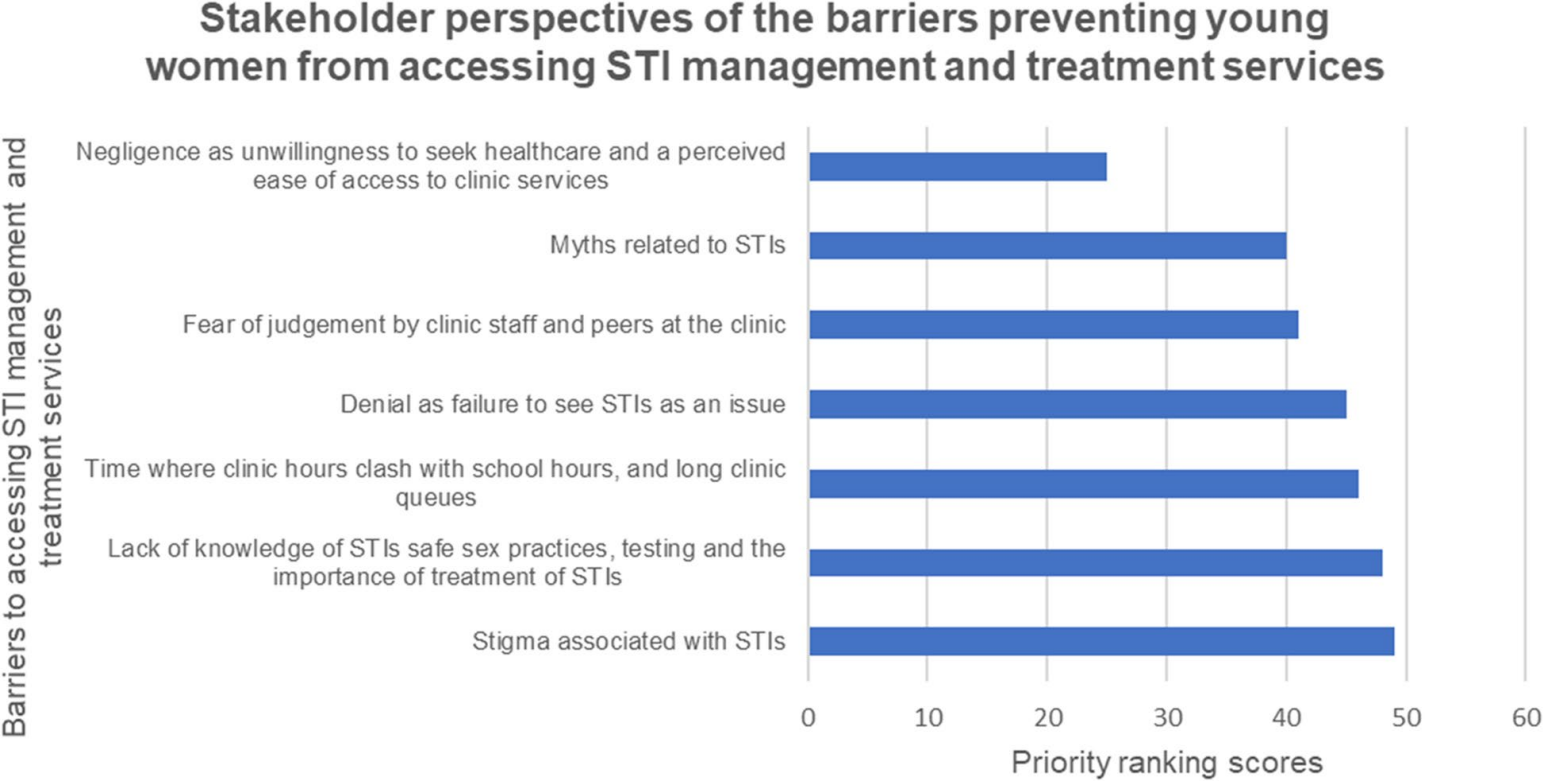
Ranking of barriers that limit access to STIs healthcare services

Stakeholders outlined the following seven factors as barriers to young women accessing STI healthcare services (Fig. 1). The priority barrier that limits or prevents young women from accessing current STI healthcare management services was the stigma associated with STIs (49 scores). This was followed by a lack of knowledge of STIs and safe sex practices (48 scores), time related to clinic hours clashing with school hours (46 scores), denial as failure to see STIs as an issue (45 scores), fear of judgement by clinic staff and peers at the clinic (41 scores), and myths related to STIs (40 scores). Negligence seen as unwillingness to seek healthcare and perceived ease of access to clinic services received the lowest priority ranking score (25 scores).

### Strategies for delivering self-sampling to diagnose STIs in young women

All eight participants were requested to suggest and rank delivery strategies to mitigate the barriers to accessing STI healthcare services, as previously outlined. Stakeholders reported the following seven strategies as idea strategies for the delivery of STI self-sampling among young women in eThekwini District Municipality (Table 2). Table 2 shows the suggested strategies in ascending order of ranking from low priority to high priority ranking. Stakeholders ranked handing out self-sampling kits at clinics, universities, schools, pharmacies or via outreach teams (98%) and educating people about the STI symptoms (98%) as the two highest priority strategies. These were followed by educating the public on how to properly use the self-sampling kits (95%), improving youth friendly services at clinics (91%), and receiving screening and testing results via SMS, email or collecting them at the clinics and then treatment being issued (88%).

**Table 2:** Ranking scores for question 2

**Delivery strategies for self-sampling for STI diagnosis**	**Summing by ranking votes** **Where***1* = *low priority* **And***7* = *high priority*							**Total number of voting scores (number of votes × ranking score)**	**Percentage of votes**
	**1**	**2**	**3**	**4**	**5**	**6**	**7**	**56**	**100%**
Self-sampling kits can be handed out at popular restaurants	1	1	2	1	1	1	1	31	55
Individuals are to collect self-sampling kits in the security guard rooms		4	1				3	32	57
Results can be received via SMS, or email or can be collected at the clinic, where treatment is received				1	2		5	49	88
Improve youth friendly services at clinics				1	1		6	51	91
Educate the public on how to properly use the kits					1	1	6	53	95
Educate people about the STI symptoms						1	7	55	98
Handout out self-sampling kits at clinics, universities, schools, pharmacies or via outreach teams						1	7	55	98

### Reported barriers versus proposed strategy

Our study results indicate that the proposed strategies by the stakeholders have the potential to address the barriers which they had highlighted. Table 3 below is an indication of the reported barriers and strategies as proposed by the stakeholders. Stigmatisation associated with STIs, lack of knowledge about symptoms of STIs, myths about STIs, and fear of judgement were reported as barriers to accessing STI healthcare services. In addition, clinic operating hours clashing with school hours, and negligence and denial by young women who may be infected with STIs, were the other barriers. The proposed strategies to mitigate these barriers include providing community education about STIs that would address the issue of stigma, judgement, and lack of knowledge about STIs. Additionally, access to diagnostic test results can be made simple by sending results using email and or SMS, in addition to being obtained from the healthcare facilities.

**Table 3:** Matching reported barriers versus proposed strategy

Reported barriers	Self-sampling delivery strategies
- Stigma (and fear of being judged for being infected)	- Educate people about the STI symptoms - Educate the public on how to properly use the kits - Improve youth friendly services at clinics - Results can be received via SMS, or email or can be collected at the clinic where treatment is received
- Lack of knowledge (myths about STIs, and negligence) - Denial	- Educate people about the STI symptoms
- Time	- Self-sampling kits can be handed out at popular restaurants - Handout out self-sampling kits at clinics, universities, schools, pharmacies or via outreach teams

### Feedback from stakeholders and suggestions for priority delivery of the strategies

The study PI compiled a report of the proceedings of the NGT which contained the proposed delivery strategies. It was shared with all 8 stakeholders, and they were requested to comment on the proposed delivery strategies for the rolling out of self-sampling for STIs as recorded in the report. It is assumed that all study participants read the report but had no additional contributions to provide. This suggested that all relevant information had been disseminated in the workshop hence no additional comments were received from the stakeholders.

### Suggested STI self-sampling priority strategies

Although barriers emerged from the discussions, it is worth noting that some of the participants had initially reported that there were no real barriers to young women accessing STI healthcare services. This implied that the STI education sessions conducted regularly at PHCs and young women having access to educational material about STIs on various media platforms, were adequate. However, since the young women still did not use the STI healthcare services provided, the participants still recommended education as a strategy to promote self-sampling as an intervention that could improve access. In addition, the stakeholders highlighted that PHCs have youth friendly services which are equipped with the necessary STI education materials which are always accessible. However, some young women were still reluctant to utilise them.

The following presents the strategies to mitigate highlighted barriers as suggested by the participants during the NGT.

Since stakeholders were placed into groups during the NGT, all of their contributions were presented per group and not as individuals. The stakeholders suggested the handing out of self-sampling kits at clinics, pharmacies, universities, and schools, or via community outreach teams to make STI healthcare services accessible.

- Group 1: “*Self-sampling kits must be readily available at pharmacies, schools, and libraries which are places where young women can be found.*
  *“Mobile clinics can hand out self-sampling kits and then collect the specimens thereafter.*
  *Community outreach teams can hand out the kits.”*
- Group 1: “*Self-sampling kits can be handed out at schools and through school clinics at TVET colleges and universities.*”
  *“Adopt condom distribution strategy for self-sampling kits to move them closer to the users.”*

The study participants highlighted that PHC operating hours clash with school and university hours:

- Group 1: “*Clinic operates from 07h30 to 16h00 but at that time most young people are still at school or college.*”
- Group 2: “*Operating hours of the clinic clash with school hours. When the clinic closes, the young people are still at school.*”

To mitigate this challenge, the participants recommended the need to address efficient provision of the youth friendly services to accommodate those who are in school. The following suggestions were made:

- Group 1: “*To shorten waiting times for young people of school going age, enforce designated adolescent youth friendly service provision at ALL clinics.*”
- Group 2: “*Ensure that adolescent youth friendly services have sufficient staffing capacity to remain open even after clinic operating hours."*

Providing education about STI symptoms was suggested as a strategy to equip young people with the necessary knowledge to identify signs and symptoms of STIs. Furthermore, it was suggested that these sessions could also be used to address some of the myths that exist about STIs:

- Group 1: “*The use of outreach campaigns to educate young people with the aid of community health workers."*
  *“Address myths and misconceptions about STIs during education sessions about STI signs and symptoms.”*
- Group 2: “*Conduct education of proper specimen collection along with health education about the signs and symptoms of STIs.*”

Considering that self-sampling is a new intervention, the participants suggested community education as a strategy. Providing education to the public on how to self-collect specimens for STI testing was ranked as a high priority strategy to ensure that specimen self-collection is done appropriately. This would be useful to ensure that self-collected specimens are of good quality and suitable for laboratory diagnostic testing of STIs:

- Group 1: “*Provide education of proper specimen collection along with health education about the signs and symptoms of STIs.*”
- Group 2: “*Patients will have to collect the self-sampling kits directly from the nurses so they can get instruction on how to use it and the turnover time for the sample.*”

Providing a youth-friendly environment at PHCs also received a high priority ranking in response to some of the barriers previously highlighted. Highlighted barriers included stigma associated with STIs which may be accompanied by judgement from clinic staff thus creating an environment that is not open and welcoming to young people. By suggesting the improvement of youth-friendly service provision at PHCs, the participants envision that would create an environment that is welcoming for young women to engage with STI healthcare services:

- Group 1: “*Adolescent and Youth Friendly Services at all healthcare facilities need to be active to improve the way young people experience STI healthcare services."*
- Group 2: “*Clinics should provide a youth friendly and non-judgemental environment for STI healthcare through designated Adolescent and Youth Friendly Services.*”

As previously highlighted by our participants, young women are afraid to disclose their participation in sexual intercourse and any symptoms they experience due to STIs and so they do not engage available STI healthcare services. The study participants suggested that after diagnosis, young women can receive STI diagnostic results via SMS, email to avoid interaction with clinic staff. However, in the event that treatment is required then results and treatment be collected from a PHC:

- Group 1: “*Come to the pharmacy or the clinic for results and treatment where treatment is required.*”
- Group 2: “*Results can be received via SMS or can be collected from the clinic to minimise interaction with clinic staff.*”

## Discussion

In this study, we aimed to explore health workers’ perspectives on a self-sampling intervention for diagnosing STIs among young women in the eThekwini District Municipality using a NGT. Through the NGT, we identified critical barriers that hinder young women from accessing STI healthcare services. Additionally, through the NGT, the healthcare workers prioritized strategies for self-sampling interventions to improve access for young women. The NGT revealed fear of judgment and the stigma associated with STIs as prominent barriers to accessing STI healthcare services. These findings align with previous research, such as the study by Cassidy et al. [30], which also highlighted stigma and fear of judgment as impediments to young people seeking care. Another study identified these same factors as deterrents for adolescent girls and young women seeking care for STI-related health issues [31]. The inability of young women to recognize STI symptoms emerged as another significant barrier, consistent with previous research that reported that young people lack knowledge about STIs and available healthcare services [32]. This lack of awareness about STIs often leads individuals to delay seeking immediate healthcare [33].

During the NGT, stakeholders suggested the following strategies for self-sampling as an intervention for STIs in women: distributing self-sampling kits at popular restaurants; making kits available in security guard rooms at PHCs; providing STI diagnostic results via SMS or email; enhancing youth-friendly services at clinics; educating the public on proper kit usage and STI symptoms; and distributing kits at clinics, universities, schools, pharmacies, or through outreach teams. The potential benefits of using these strategies in healthcare have been well documented. Notably, education is one of the key strategies to facilitate the link between available public services and the communities they are meant to serve [34]. Additionally, a study by Wang et al. [35] established that health education not only contributes to knowledge but also practices towards infectious diseases, which has the potential to improve healthcare seeking behaviour. However, it’s crucial to emphasize while knowledge about STIs may confer precaution, it may not necessarily reduce risk-taking behaviour [36]. The sub-Saharan, has experienced rapid growth in the use of smartphones and the internet [37], thus offering a great potential for eHealth interventions to revolutionise healthcare interventions. The idea of patients receiving their STI diagnostic results via SMS or email thus aligns with the technological advancements. Several studies have proven that adopting eHealth opportunities can transform the user perceptions and accessibility of STI healthcare services, especially in LMICs [38–40].

### Strengths and limitations

The utilisation of NGT fostered collaboration among healthcare service providers enabling the development of key strategies for self-sampling for STI diagnosis among women. True to NGTs, this collaborative approach resulted in a wide range of views which enhanced the comprehensiveness of the strategies to improve access to STI healthcare services. The use of NGTs to promote collaboration and generation of diverse perspectives in well known [25]. Using NGT limited the bias of having one dominant participant by utilising ranking to identify high priority strategies. This approach ensured that the most critical strategies were identified based on consensus rather than individual influence. The use of NGT to eliminate bias and promote consensus has been well proven and established over time, dating back to Delbecq et al. in 1975 to date [28, 41].

Due to the nature of PHCs having limited medical personnel and being understaffed, most of the stakeholders who participated in the NGT were nurses involved in STI healthcare services. Due to the scarcity of medical doctors at PHCs, no medical doctor was available to participate in the NGT on the day that it was conducted. The limited representation of medical doctors due to staffing constraints at PHCs is a challenge recognised by the World Health Organization [42]. Although nurses and medical doctors work hand in hand to provide healthcare services to patients, there is a vast difference in the academic training received. The academic training of and duties of medical doctors is different from that of nurses. Medical doctors often have in-depth clinical expertise in diagnosing and treating STIs and so their participation could have enhanced the knowledge gathered and strategies developed. Additionally, the determination of the level of acceptability of the suggested delivery strategies by young women was not evaluated.

## Conclusions

Our study identified key barriers and strategies for implementing self-sampling interventions for STIs in young women. These findings highlight the need to address stigma, fear of judgment, and provide comprehensive education to improve healthcare-seeking behaviour in young women. Furthermore, the study also indicates that embracing eHealth solutions could enhance the accessibility and efficiency of STI healthcare services in LMICs significantly. Our findings have the potential to influence policy changes regarding the provision of STI healthcare services for young women. Prior to the implementation of these strategies, a similar study to co-create strategies for self-sampling in young women in collaboration with young women is required. This proposed follow-up study is likely to foster the development of a more patient-tailored approach to the management of STIs within this population.

## Data Availability

All data generated or analysed during this study are available in the manuscript. However, additional datasets used are available from the corresponding author on reasonable request if required.

## Abbreviations

DCE: Discrete Choice Experiment
HIC: High-income countries
LMIC: Low-and-middle income countries
NGT: Nominal Group Technique
PHC: Primary Healthcare Clinic
PI: Principal Investigator
TVET: Technical and Vocational Education and Training
